# Benchmarking European Home Care Models for Older Persons on Societal Costs: The IBenC Study

**DOI:** 10.1177/11786329211022441

**Published:** 2021-06-21

**Authors:** Lisanne I van Lier, Henriëtte G van der Roest, Vjenka Garms-Homolová, Graziano Onder, Pálmi V Jónsson, Anja Declercq, Cees MPM Hertogh, Hein PJ van Hout, Judith E Bosmans

**Affiliations:** 1 Departments of General Practice & Medicine of Older Persons, Amsterdam Public Health research institute, Amsterdam UMC, Vrije Universiteit Amsterdam, Amsterdam, the Netherlands; 2 Department on Aging, Netherlands Institute of Mental Health and Addiction (Trimbos Institute), Utrecht, the Netherlands; 3 Department III, Economy and Law, Hochschule für Technik und Wirtschaft Berlin, University of Applied Sciences, Berlin, Germany; 4 Department of Cardiovascular, Endocrine-metabolic Diseases and Aging Istituto Superiore di Sanità, Rome, Italy; 5 Department of Geriatrics, Landspitali University Hospital, and Faculty of Medicine, University of Iceland, Reykjavik, Iceland; 6 LUCAS, Centre for Care Research and Consultancy, and CESO, Center for Sociological Research, KU Leuven (University of Leuven), Leuven, Belgium; 7 Department of Health Sciences, Faculty of Science, Amsterdam Public Health research institute, Vrije Universiteit Amsterdam, Amsterdam, the Netherlands

**Keywords:** Home care models, societal costs, older adults, international benchmarking

## Abstract

This study aims to benchmark mean societal costs per client in different home care models and to describe characteristics of home care models with the lowest societal costs. In this prospective longitudinal study in 6 European countries, 6-month societal costs of resource utilization of 2060 older home care clients were estimated. Three care models were identified and compared based on level of patient-centered care (PCC), availability of specialized professionals (ASP) and level of monitoring of care performance (MCP). Differences in costs between care models were analyzed using linear regression while adjusting for case mix differences. Societal costs incurred in care model 2 (low ASP; high PCC & MCP) were significantly higher than in care model 1 (high ASP, PCC & MCP, mean difference €2230 (10%)) and in care model 3 (low ASP & PCC; high MCP, mean difference €2552 (12%)). Organizations within both models with the lowest societal costs, systematically monitor their care performance. However, organizations within one model arranged their care with a low focus on patient-centered care, and employed mainly generalist care professionals, while organizations in the other model arranged their care delivery with a strong focus on patient-centered care combined with a high availability of specialized care professionals.

## Background

In Europe, the proportion of older adults aged 65 years and over is expected to grow from 32% in 2020 to 52% in 2050.^1,2^ This will lead to a high demand for long term care services, since ageing is strongly associated with multimorbidity, limitations in daily activities, and higher dependency in general.^3,4^ It is unlikely that this projected increase in the demand for intensive long term care by older adults in a health care system in its current form will remain affordable. The main cost drivers of care for older adults currently are long term institutional care and hospitalization.^5^ Therefore, decision makers seek ways to restrain the rising expenditures on long-term care by substituting institutionalized care with home-based care, since the costs of home care are generally assumed to be lower than costs of institutionalized care.^6^ As a consequence, diverse EU policies promote home care for older adults.^3,7^ Home care is also preferred by most older adults themselves, as they value their independence.^8^

Most European countries now offer a wide range of home care services for older adults, including home nursing care, personal care, social care, and specific treatments (eg, physical therapy).^9-11^ However, the availability of home care, and the way in which home care is provided varies within and between counties.^5,12^ Care models are a useful multidimensional concept to define the way care is delivered, also referred to as ‘a descriptive picture of practice which adequately represents the real thing’.^13^ Care models can distinguish between care practices with regard to, for example, the provision of social care services and the level of working arrangements in- and outside the organization.^10^ Other home care models described in the literature focus on case management, integrated care, consumer-directed care, or restorative care.^14-16^

Care delivery arranged according to different home care models may lead to differences in resource utilization and costs of care. Reviews reported that coordinated care, integrated care, case management, and consumer-directed care were found to delay nursing home admissions and decrease hospital use, but increased home-based service use.^14,15,17,18^ The results on costs, however, were inconclusive.^14,15,17-20^ When studying costs of care delivery, only a few of the included studies considered societal costs (costs of health, social and informal care). Especially in home care for older adults, this perspective is important, because it provides a comprehensive view of costs, while also showing shifts in the distribution of cost across cost categories. Insight into these shifts is necessary due to the increased relevance and provision of informal care and welfare support in the care of older adults with chronic disorders.

Understanding whether and how the organization of home care is related to societal costs may help policymakers and organizations to achieve costs reductions. To the best of our knowledge, no head-to-head comparisons of different home care models on societal costs have been performed previously. Therefore, the aim of this study was to benchmark societal costs (healthcare, home care and informal care costs) per client in different home care models and to describe characteristics of home care models with the lowest societal costs.

## Methods

### Design

This study has a prospective longitudinal design, with assessments at baseline, 6 and 12 months. The study is part of the cross-European ‘Identifying best practices for care-dependent elderly by Benchmarking Costs and outcomes of community care’ (IBenC) project, funded within the 7th Framework Program of the European Commission. IBenCs primary aim is to identify best practices in home care models across Europe by comparing their costs and quality of care outcomes.^21^ The study was approved by relevant legally authorized medical ethical committees in the countries that participated in the IBenC project.

### Setting and participants

Thirty-eight home care organizations from 6 countries, Belgium, Finland, Germany, Iceland, Italy, and the Netherlands, participated in the IBenC project. The IBenC sample consisted of community-dwelling adults aged 65 years and older receiving home care from a (primary care) nurse, and expected to remain in care for at least 6 months after inclusion.

### Procedure

Professional care organizations that offer health or social care to people in the community were invited by the national study centers to participate in the study. Participation of organizations already using the interRAI-HC in their care practice was preferred. Diversity between community care organizations regarding structures, size, and type of region (urban, rural, or mixed) was sought. After enrolment of care organizations, eligible clients were selected from the organizations’ caseload. For participants from some organizations in The Netherlands and Italy that used the interRAI-HC in routine care practice, informed consent for the study was not required according to local regulations. All other clients signed an informed consent form before entering the study.

Information on client characteristics, health outcomes, and care utilization was collected at baseline, and after 6 and 12 months between 2013 and 2016. The assessments were conducted in the homes of the care recipients by trained (research) nurses. The questionnaire on organizational characteristics was completed by a key person of each of the participating care organizations.

### Home care models

**Figure 1.**
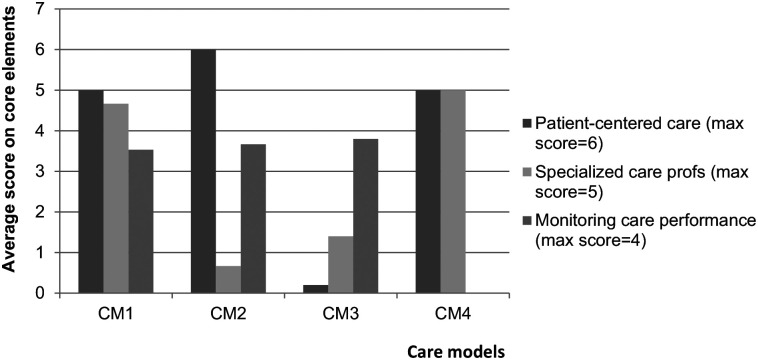
Composition of the identified home care models according to core elements of care delivery. Abbreviation: CM, care model. Higher average scores indicate higher focus on the core element.

Six different home care models were distinguished based on information on organizational characteristics collected within the project. By means of a cross-sectional questionnaire that was designed specifically for use in the IBenC study,^22^ information was collected on structural features, management, care processes, staff characteristics, and reimbursement systems. From these data, 3 core elements of care practice arrangements emerged: the level of patient-centred care, the availability of specialized care professionals, and the level of monitoring of care performance.^22^ Patient-centred care was operationalized using 6 items referring to elements of patient-centered care delivery such as actively involving clients and informal caregivers in care planning, or the availability of a client’s (digital) file.^22,23^ The availability of specialized care professionals was assessed using 5 items on employment of care professionals in specialized care domains, such as dementia care or palliative care. The third element, level of monitoring of care performance, refers to the standardized assessment of quality of care and client satisfaction on received care and was operationalized by 4 items. Organizations were classified into 6 distinct home care models according to the level to which their care arrangement met each of the core elements.^22^ Higher scores on an element indicate a higher focus on this core element of their care arrangement (see Figure 1). Care model 1 (CM1) is characterized by a (very) strong focus on patient-centred care provided by specialized care professionals in which the performance of care is monitored closely. Care model 2 (CM2) is characterized by close monitoring of the performance of care that is very much patient-centered, but with little or no availability of specialized care professionals. Care model 3 (CM3) focuses on monitoring of care performance, with less focus on patient-centered care and employment of specialized care professionals. Care model 4 (CM4) consists of patient-centered care that is provided by specialized care professionals, but monitoring of care performance is not routinely done. More details on the care models are included in the paper by Van Eenoo et al.^22^

Two of the 6 care models were excluded for this study (CM5 and CM6), because only 2 organizations with a low number of respondents (n = 15 and n = 17) delivered care according to these models.

### Measures

#### Client characteristics

To assess baseline client characteristics, the interRAI-Home Care (interRAI-HC) instrument was used. The interRAI-HC is a standardized and reliable comprehensive geriatric assessment instrument designed to assist in care planning, outcome measurement, quality improvement, and resource allocation for clients who receive care at home.^24-27^ The instrument contains several validated outcome scales. Cognitive functioning is assessed by the Cognitive Performance Scale (CPS, range 0-6). Moderate to severe cognitive impairment is considered to be present if the CPS score is 3 or higher.^28^ Depressive symptoms are assessed by the Depression Rating Scale (DRS, range 0-14). A score of 3 or higher on the DRS indicates the possible presence of minor or major depressive disorder.^29^ Activities of daily living (ADL) needs were assessed with the interRAI Activities of Daily Living Hierarchy Scale (ADLH, range 0-6).^30^ Difficulties in performing instrumental activities (iADL) were assessed using the Instrumental ADL Performance Scale (iADLP, range 0-48).^31^ Medical complexity/health instability was assessed using the Changes in Health, End-Stage Disease, Signs, and Symptoms Scale (CHESS, range 0-5).^32,33^ Higher scores on the abovementioned scales reflect higher complexity and care needs.

#### Care utilization

Health and social care utilization and informal caregiver time were assessed with the resource utilization items from the interRAI-HC at baseline and 6-month follow-up. The interRAI-HC was considered a valid instrument to assess the use of care services from a societal perspective.^34^ The number of events or days and the total number of minutes of care received were registered. Recall periods were 90 days for hospitalization, emergency room and physician visits, 7 days for regular home care services and visits to therapists, and 3 days for informal care. Care utilization estimates (number of events, amount of time) were divided by their recall period in days, and multiplied by 91 days to reflect a period of 3 months. The interRAI-HC assesses the number of hospital stays, but does not assess the number of nights (except for Belgium). To estimate the number of nights, we multiplied the reported number of events with country-specific averages of length of stay during hospital admission in the year 2012^35^ (Appendix 1). In Belgium, the registered total number of hospital admission days was used instead of OECD estimates.

#### Dependent variable

Societal costs of care utilization over a 6-month period were calculated by multiplying resource utilization with Dutch standard costs.^36^ Cost of informal care was estimated by multiplying informal care hours with the wage rate of a legally employed cleaner.^36^ Using uniform costs ensures that differences between care models are not influenced by country specific price differences. For institutionalized or deceased respondents, we assumed that the event occurred halfway between the 2 assessments. Costs after admission to another care setting were calculated using standard cost per day for the specific facilities, and costs after death were determined to be zero.

Seven cost categories were distinguished (Appendix 1) and summed into total societal costs. To calculate the societal costs over a 6-month period, costs between assessments were linearly interpolated by multiplying costs at baseline assessment by 0.5, and costs at 6-month follow-up were multiplied by 1.5. Thus costs in the first 3 months were based on the average of the costs at baseline assessment and 6-month follow up, while costs in the seconds 3 months were based on the 6-month assessment.

### Analysis strategy

All analyses were performed using SPSS statistics 20 and STATA 12 SE. Baseline characteristics were described using descriptive statistics and frequencies. Differences in baseline characteristics between participants from different home care models, and between participants who dropped out and participants who remained in the study were evaluated using Chi-square tests for categorical variables and analysis of variance (ANOVA) tests for continuous variables. The significance level was set at 0.05 in all analyses.

Missing cost data at 6 months were imputed using multiple imputation with chained equations in SPSS.^37^ Predictive mean matching was used to account for the skewed distribution of costs. Baseline characteristics that were significantly associated with costs after 6 months, differed significantly between home care models, or between respondents with and without follow-up were included in the imputation model. Ten datasets were created. Each imputed dataset was analyzed separately, and the results of the analyses were pooled using Rubin’s rules.^38^

The amount of informal caregiving time was not assessed in Belgium. Therefore, CM4 (Belgian organizations only) was not included in the main analysis from a societal perspective. Mean disaggregated costs and total societal costs were calculated per client over a 6-month period stratified for each care model. Differences in costs per client between home care models were analyzed using linear regression with dummy variables for the home care models. Because of the skewed distribution of cost data, 95% confidence intervals (CIs) were estimated using bias-corrected accelerated bootstrapping with 5000 replications. Differences were adjusted for the following case mix variables: age, sex, living status, CPS, DRS, ADLH, IADL, and CHESS. Also country of residence was explored as potential confounder. Collinearity between covariates was investigated using Spearman rho correlation coefficients (cut-off value r > 0.4).

Two sensitivity analyses were performed. First, an analysis was performed from a healthcare perspective (excluding informal care costs) including Belgian participants (16 organizations, n = 493). Two Belgian organizations (n = 32) were excluded since they were assigned to the 2 care models that were not considered in this study (n too small). Second, to evaluate the robustness of the valuation method on the results, units of resource utilization were multiplied by Italian prices^39-41^ indexed to the year 2015, which considerably deviated from the Dutch standard costs (Appendix 1).

## Results

A total of 2060 participants in 3 care models from the original sample (n = 2884) were included in the main analyses. Data from 824 respondents were excluded from the main analysis; because of software problems data collection of interRAI-HC was put on hold in 1 Dutch organization (n = 224) and uncollected information on informal care hours in Belgium (18 organizations, n = 525). In addition, 2 German organizations could not be assigned to a care model due to incomplete data (n = 75). CM1 contained 6 organizations and 1331 participants (65%), CM2 respectively 3 and 311 (15%), and CM3 9 organizations and 418 participants (20%). See Table 1.

**Table 1.** Characteristics of the study population per care model.

	Societal perspective (including informal care)					Healthcare perspective (without informal care)					
	CM1 n = 1331	CM2 n = 311	CM3 n = 418	Total N = 2060	*P*-value	CM1 n = 1716	CM2 n = 311	CM3 n = 439	CM4 n = 87	Total N = 2553	*P*-value
Number of organizations	6	3	9	18	–	15	3	10	6	34	–
Country (n, %)	IT 499 (37%) NL 111 (8%) IS 420 (32%) FI 301 (23%)	NL 156 (50%) FI 155 (50%)	GE 418 (100%)	IT 499 (24%) NL 267 (13%) IS 420 (21%) FI 456 (22%) GE 418 (20%)	–	IT 499 (29%) NL 111 (6%) BE 385 (22%) IS 420 (24%) FI 301 (18%)	NL 156 (50%) FI 155 (50%)	BE 21 (5%) GE 418 (95%)	BE 87 (100%)	IT 499 (20%) NL 267 (10%) BE 493 (19%) IS 420 (16%) FI 456 (18%) GE 418 (16%)	–
Mean age (SD)	82.7 (7.5)	82.4 (7.2)	83.5 (7.7)	82.8 (7.5)	.07	82.7 (7.3)	82.4 (7.2)	83.5 (7.6)	82.2 (7.3)	82.8 (7.3)	.10
Female (n, %)	874 (66%)	209 (67%)	295 (71%)	1378 (67%)	.18	1125 (66%)	209 (67%)	311 (71%)	59 (69%)	1704 (67%)	.22
Living alone (n, %)	675 (51%)	225 (73%)	310 (74%)	1210 (59%)	<.01	844 (49%)	225 (72%)	325 (74%)	53 (61%)	1447 (57%)	<.01
Cognitive impairment (CPS ⩾ 3) (n, %)	255 (19%)	17 (6%)	117 (28%)	389 (19%)	<.01	323 (19%)	17 (6%)	118 (27%)	12 (14%)	470 (19%)	<.01
Depressive symptoms (DRS ⩾ 3) (n, %)	159 (12%)	49 (16%)	82 (20%)	290 (14%)	<.01	234 (14%)	49 (16%)	87 (20%)	10 (12%)	380 (15%)	<.01
Mean ADLH score (SD)	1.9 (2.1)	0.6 (1.3)	2.2 (1.8)	1.7 (2.0)	<.01	2.2 (2.0)	0.6 (1.3)	2.3 (1.8)	2.6 (1.4)	2.0 (1.9)	<.01
Mean iADLH score (SD)	30.6 (14.1)	23.0 (12.4)	29.1 (15.0)	29.2 (14.3)	<.01	31.5 (13.5)	23.0 (12.4)	29.3 (14.8)	30.0 (11.9)	30.1 (13.8)	<.01
CHESS (SD)	1.2 (1.1)	0.8 (0.9)	0.6 (0.9)	1.0 (1.1)	<.01	1.1 (1.1)	0.8 (0.9)	0.6 (0.9)	0.8 (1.0)	0.9 (1.1)	<.01
Informal caregiver expresses feelings of distress, anger or depression (n, %)	227 (18%)	27 (10%)	24 (10%)	278 (15%)	<.01	277 (17%)	27 (10%)	25 (10%)	6 (8%)	335 (15%)	<.01
Mean number of hours home care per week at baseline (SE)	2.2 (0.1)	4.4 (0.3)	6.8 (0.3)	3.5 (0.1)	<.01	3.3 (0.1)	4.4 (0.3)	6.9 (0.3)	4.6 (0.5)	4.1 (0.1)	<.01
Mean number of hours informal care per week at baseline (SE)	30.4 (1.1)	16.4 (1.9)	11.5 (1.3)	24.5 (0.8)	<.01	–	–	–	–	–	

Abbreviations: ADLH, Activities of Daily Living Hierarchy Scale; CHESS, Changes in Health, End-Stage Disease, Signs, and Symptoms Scale; CM, care model; CPS, Cognitive Performance Scale; DRS, Depressive Rating Scale; FI, Finland; GE, Germany; iADLH, instrumental ADL Performance Scale; IS, Iceland; IT, Italy; NL, Netherlands.

In total, 29% of the participants had missing values on one or more resource utilization items. The amount of missingness was 0% for costs of institutionalized care, and 6% to 7% for the other cost categories.

Two-thirds of the participants in the study sample were female, and the mean age was 82.8 years (SD = 7.5) (*P* > .05). Participants in CM1 lived less often alone (49%), received the largest amount of informal care at baseline (30.4 hours per week, SD = 1.1), and the frequency of caregiver distress was higher as compared to the other CMs (*P* < .01). In CM2, participants were least impaired in cognition, only 6% experienced moderate to severe cognitive impairment, and in (i)ADL (*P* < .01). CM3 contained participants with the highest impairments in cognition (28% moderate to severe cognitive impairment) and (i)ADL, and had the highest rate of depressive symptoms (20%) as compared to participants in the other CMs (*P* < .01). They also received the largest amount of professional home care per week at baseline compared to participants in the other CMs (6.8 hours, SD = 0.3) (*P* < .01). See Table 1.

Between the baseline and 6-month follow up assessments, 3% (n = 91) of the participants were admitted to a nursing home, hospital, or rehabilitation facility, 2% (n = 71) died, <1% (n = 14) were discharged from home care, and 2% of the participants were lost for follow-up due to lack of interest or time (n = 16) or without reason (n = 37). In CM3, 7% of the participants died, which was higher compared to the other CMs (1%-3%) (*P* < .05). Compared to the completers, the drop-outs were statistically significantly older (*P* < 0.05).

**Figure 2.**
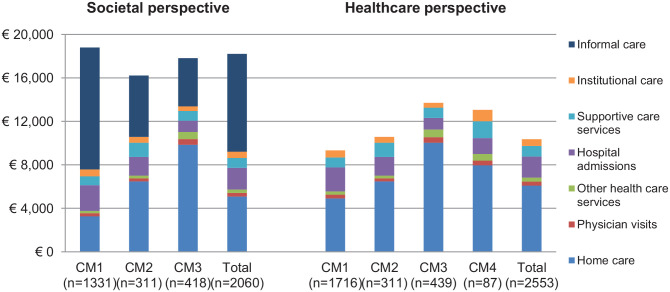
Six-month cost of care estimates per client not adjusted for case mix differences across home care models. Abbreviation: CM, care model.

With average costs per client of €16221 (SE = 854) over 6 months, mean unadjusted total societal costs per client were lowest in CM2 and highest in CM1 (€18800, SE = 404) (Figure 2). In CM1, approximately 60% of the total societal costs was attributed to informal care, this share was much lower in CM3 (25%) and CM2 (35%). In CM3, home care accounted for approximately 54% of total societal costs, which was considerably higher compared to the other care models. CM3 generated relatively few hospitalization costs (6% of total share), while this share was almost double for CM1 and CM2. Costs of physician visits, other healthcare services, supportive care services, and institutional care only contributed marginally to the total societal costs. See Appendix 2 and 3 for more details.

**Figure 3.**
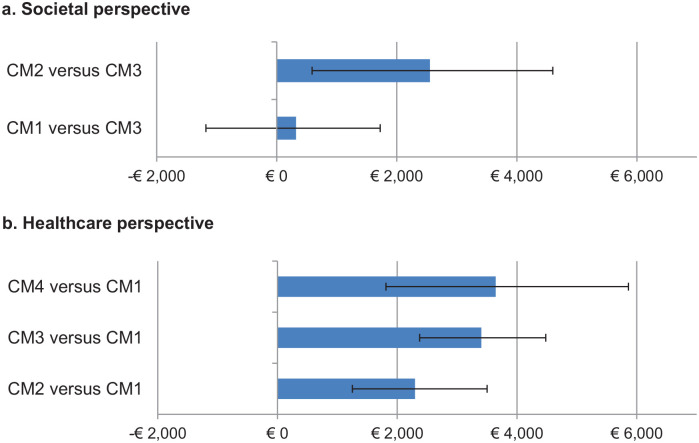
Mean adjusted differences in total societal (a) and healthcare (b) costs per client between the care models. Abbreviation: CM, care model. In the comparisons, the care model with the lowest costs act as reference. 95% confidence intervals were estimated using bias-corrected and accelerated bootstrapping with 5000 replications. Significant differences in costs between a care model and the reference care model can be considered when the confidence interval do not cross the €0 costs line. Significant differences in costs between care models can be considered when their confidence intervals do not overlap.

After adjusting for case mix, mean total societal costs per client in CM2 were statistically significantly higher than in CM1 (mean difference €2230, 95% CI 551; 4179) and in CM3 (mean difference €2552, 95% CI 588; 4599) (Figure 3a). Differences in mean total societal costs per client between CM1 and CM3 were not statistically significant (mean difference €321, 95% CI −1180; 1723). Country of residence did not explain any additional variance and was not included as a covariate.

### Sensitivity analyses

A sensitivity analysis was performed from a healthcare perspective including Belgian participants (16 organizations, n = 493). Belgian participants received care that was mostly provided according to CM1 (n = 385, 78%) and CM4 (n = 87, 18%), with a smaller proportion receiving care according to CM3 (n = 21, 4%). After including Belgian participants, a lower proportion of the participants in CM1 lived alone and relatively more experienced depressive symptoms. Also, slightly higher rates were found for (i)ADL impairment and the amount of professional home care. Baseline characteristics of participants in CM3 hardly differed. Participants in CM4 had more ADL limitations (2.6, SD = 1.40) as compared to participants in the other CMs (*P* < .01) (See Figure 1 and Appendix 2).

Mean unadjusted total healthcare costs per client were lowest in CM1 (€9333, SE = 221) and highest in CM3 (€13701, SE = 533). CM1 was associated with the lowest share (as proportion of total healthcare costs) for home care (53%) and the highest share for hospital admissions (24%), as compared to the other CMs. In CM3, home care accounted for approximately 73% of total healthcare costs, which was considerably higher than in the other CMs. In CM4, 61% of total healthcare costs could be attributed to home care and 11% to 12% to hospital admissions and supportive care.

After adjustment for case mix variables, as compared to the other care models, CM1 was associated with statistically significantly lower healthcare costs per client than in CM2 (mean difference €−2297, 95% CI (−3510; −1242), CM3 (mean difference €−3405, 95% CI (−4484; −2385), and in CM4 (mean difference €−3644, 95% CI (−5898; −1826). CM4 was associated with the highest healthcare costs per client, but differences between this model and CM2 and CM3 were not statistically significant (Figure 3b).

In the second sensitivity analysis, 6-month societal costs were estimated using Italian prices. The results were comparable, although cost differences across models were less pronounced. CM2 was still associated with statistically significantly higher societal costs per client than CM1 (mean difference €779, 95% CI 98; 1581) and CM3 (mean difference €1072, 95% CI 265; 1935). Total societal costs per client in CM3 were not significantly lower than those in CM1 (mean difference €−274, 95% CI −865; 338).

## Discussion

To the best of our knowledge, this is the first study in which home care models in a large European sample were compared on the basis of societal and healthcare costs. We found that 2 distinct home care models (CM1 and CM3) were associated with statistically significantly lower societal costs per client as compared to the third care model (CM2). Also, the ratio of home care costs to informal care costs differed considerably between these 2 care models. Of the 2 care models with the lowest societal costs, 1 model was characterized by a low focus on patient-centred care and employment of mainly generalist care professionals, which resulted in relatively high home care costs and low informal care costs (CM3, ratio 1:0.5). In contrast, the other had a strong focus on patient-centered care in combination with high availability of specialized care professionals, and relatively low home care costs and high informal care costs (CM1, ratio 1:3), implying that only a small part of day-to-day care is provided by professionals. Both models also focused on monitoring of care performance.

In light of the current reforms of long-term care to limit care expenses, the ratio of professional home care to informal care in CM1 seems more favorable than that in CM3. The reforms aim at a reduction in the intensity of professional home care in combination with strengthening the involvement of informal caregivers. As a consequence, families are facing increased responsibilities for providing care in old age.^42^ It is questionable whether this situation is fully desirable as, for example, costs of hospital admissions were relatively high in CM1, as was caregiver distress. Research shows that caregiver distress is related to increased healthcare utilization and lost productivity in informal caregivers.^43^ In CM3 lower levels of caregiver distress were reported, even though the clients in this model had more severe impairments at baseline than those in CM1. So, possibly the amount of professional home care in CM1 is insufficient, resulting in adverse effects, such as hospitalization of clients and overburdening of informal caregivers. These results highlight the importance of early identification of persons ‘at-risk’ of hospitalization and caregiver distress in situations where relatively little professional home care is provided in relation to informal care.

CM2, which was associated with the highest societal costs, differs from the other care models by its low availability of specialized care professionals combined with a relatively strong focus on both patient-centered care and monitoring of care performance. The ratio of home care costs to informal care costs was 1:2. Interesting here is that the highest adjusted societal costs were generated by this model, while the dependency levels of the clients served through this model were relatively the lowest, implying that, on average, clients received more care regardless of their health status as compared to the other models. As such, the efficiency of the care provided in CM2 is suboptimal.

### Considerations

An advantage of classifying the care as provided by care organizations into care models to study efficiency, is that a variety of different organizational elements of home care organizations regarding structure, management, care processes, staff characteristics, reimbursement systems are reduced to a number of relevant core domains that define the way care is delivered.^12^ This makes it easier to study care practices and their costs, as this enabled us to group home care clients into our analyses. However, the number of respondents in 2 of 6 care models was too small to include in our analyses. Therefore, we may have missed relevant costs differences between care models due to power issues. Also, as the care models that were included in the main analysis only differed on 2 of the 3 core elements, it was difficult to draw concrete conclusions on the relation between the elements that formed the care models and societal costs.

International benchmarking, as was done in this study, is useful in trying to find innovative and alternative ways of providing (home) care services in responding to the problem of rising expenditures on long-term care.^44^ Especially when an organization is providing a unique service and there are no organizations within the country that to benchmark with, international benchmarking can bring important added value. Further, it may reveal more differences in performance than if the comparison is only done within a country. Usually more divers practices will be found if organizations are compared across a number of countries. To increase diversity even more, we purposefully sampled diverse home care organizations and classified them into different care models. Although international benchmarking has clear benefits, it is also a complicated type of benchmarking as there is a transfer-problem.^45^ A simple transfer of care models that are associated with low societal costs is complicated by different national structures and societal preferences.^46^ Also, we cannot be sure that results will be the same if other patient groups receive care according to the care models defined. Nevertheless, if it appears that a particular care model leads to better care against lower societal costs, care organizations should be encouraged to align their care with this care model, while closely monitoring their performance.^12^

In international costing studies, it is recommended to use country-specific standard unit costs to value health services as this approaches societal opportunity costs.^47^ The application of standard unit costs enables a meaningful comparison of costs differences that result from the different care practices.^48,49^ This way cost differences can be attributed to differences in resource utilization, rather than to differences in costing methodology.^50,51^ Standard unit costs were not available for all services for all countries under study. Instead, we used Dutch standard costs in the main analysis and Italian standard costs in a sensitivity analysis. Consequently, we were able to provide a relative benchmark of societal costs and healthcare costs across care models. The disadvantage of using this approach, is that costs do not reflect actual care costs within the included countries.

Several approaches can be used to adjust for case-mix differences at baseline in observational studies. The most traditional approach to handle this is covariate adjustment using regression modelling. Commonly used alternatives include matching or balancing techniques to match similar individuals in the comparison groups with the aim to achieve a balance in covariates across groups, for example by using propensity scores.^52-54^ These alternative approaches have theoretical advantages over covariate adjustment. However, studies comparing the performance of covariate adjustment with these alternative approaches did not show that these alternative approaches are necessarily superior to covariate adjustment.^55-57^ Since matching becomes more complicated when there are more different groups, we decided to use multivariate regression analysis to adjust for case mix differences.

### Strengths and limitations

This study is the first to benchmark costs associated with different home care models. Another strength of this study is that a large number of organizations and clients from 6 European countries were included in the analyses, enabling comparison of costs between different care models. Care models were classified according to detailed information of participating home care organizations. The care model core elements together explained a substantial and sufficient proportion (75.4%) of the variance within organizational characteristics.^22^ Another strength is that the interRAI-HC assessments are reliable and valid sources of clinical functioning as well as of formal and informal resource use.^24,34^ Uniform data collection using a valid instrument greatly facilitated reliable cross-national societal cost comparisons at an organizational level, including case mix adjustment. Using routine care data for cost of care assessments keeps the burden for participants and care organizations low. Furthermore, differences in societal costs were robust for relatively different nations’ prices/tariffs, since the initial conclusions of the study did not change when resource utilization was valued with Italian standard costs.

Distinct differences between the care models that were included in the main analysis were limited, which can be a potential limitation of the study. We could not include 2 of the 6 identified care models, because only 2 organizations with a low number of respondents delivered care according to these models. Further, client groups between care models differed considerably. We adjusted for case mix characteristics to account for this. Another limitation is that the exact length of hospital stays was not recorded in the interRAI-HC (except for Belgium) and was estimated using averages from the Organization for Economic Cooperation and Development (OECD), resulting in 2 different approaches that were used to value hospital stays.^35^ Finally, participants were lost to analysis due to software omissions, and informal caregiver time was not registered in Belgium. Most Belgian clients received care according to CM4, which was totally excluded in the main analysis, while a small percentage received care according to CM1 and CM3.

## Conclusions

Results from international benchmarking of home care models on costs of resource utilization provide insights in the costs resulting from different home care practices. All 3 care models that were included in the main analysis had a strong focus on monitoring care performance. Two opposite models were associated with the lowest societal costs. One was further characterized by a low focus on patient-centered care and employment of mainly generalist care professionals, while the other had a strong focus on patient-centered care in combination with a high availability of specialized care professionals. Therefore, it was difficult to draw definite conclusions on the relation between the core elements of the care models and societal costs. Our results suggest that low societal costs can be achieved in different ways which can have more or less favorable outcomes for clients or informal caregivers. Future benchmarking studies of home care models on societal costs should focus on the contribution of home care costs and informal care costs to societal costs, as the ratio of home care to informal care differed considerably between the models. Considering current reforms of long-term care in many European countries, the ratio of home care to informal care in the model with a strong focus on patient-centered care in combination with a high availability of specialized care professionals seems more favorable than the one in the other model with lowest societal costs. Early identification of persons ‘at-risk’ of hospitalization and caregiver distress might be important focus areas for home care organizations that provide relatively little home care in relation to informal care.

## Appendix

**Appendix 1.** Overview of cost categories with associated unit costs (in €2015) and average length of stay (days).

Care service		Dutch costs^36^	Italian costs^39-41^*
Home care			
Home health aide (per hour)		€50	€18
Home nursing (per hour)		€73	€24
Physician visits			
General practitioner visit/outpatient clinic visit (per visit)		€92	€57
Other healthcare services			
Physical therapy (per session)		€33	€26
Occupational therapy (per session)		€34	€26
Social worker (per session)		€64	€22
Hospital admissions			
Hospital admission with overnight stay (per day with overnight stay)		€479	€405
Average length of hospital stay^35^	Days		
Finland	11.0		
Germany	9.2		
Iceland	5.8		
Italy	7.7		
Netherlands	5.2		
Emergency room visit without overnight stay (per visit)		€261	€242
Supportive care services			
Home making services (per hour)		€23	€18
Meals on wheels (per day)		€7.50	€6.8**
Institutionalized care			
Nursing home (per day)		€168	€152**
Rehabilitation institute (per day)		€460	€417**
Informal care			
Informal care (per hour)		€14.08	€3.75

* Italian prices were used for sensitivity analysis.

** Not available, Dutch standard costs were converted into Italian prices using Purchasing Power Parities (PPP).

**Appendix 2.** Unadjusted cost of care estimates across home care models over 6 months.

Cost category	Societal perspective (including informal care)				Healthcare perspective (without informal care)				
	CM1	CM2	CM3	Total	CM1	CM2	CM3	CM4	Total
	n = 1331	n = 311	n = 418	N = 2060	n = 1716	n = 311	n = 439	n = 87	N = 2553
	Mean (SE)	Mean (SE)	Mean (SE)	Mean (SE)	Mean (SE)	Mean (SE)	Mean (SE)	Mean (SE)	Mean (SE)
Home care	3262 (140)	6479 (426)	9844 (468)	5084 (160)	4910 (171)	6479 (426)	10 031 (460)	7952 (835)	6086 (157)
Physician visits	284 (11)	274 (30)	526 (22)	332 (10)	342 (14)	274 (30)	510 (22)	435 (62)	366 (11)
Other healthcare services	209 (16)	249 (37)	652 (59)	305 (17)	305 (19)	249 (37)	705 (60)	613 (148)	377 (18)
Hospital admissions	2369 (106)	1730 (229)	1034 (138)	2002 (83)	2207 (116)	1730 (229)	1072 (142)	1462 (454)	1928 (87)
Supportive care services	815 (33)	1300 (88)	898 (47)	905 (27)	913 (31)	1300 (88)	940 (48)	1546 (180)	986 (26)
Institutional care	640 (88)	541 (160)	429 (147)	582 (68)	657 (77)	541 (160)	444 (144)	1054 (418)	620 (62)
Total healthcare costs	7580 (193)	10 574 (554)	13 384 (535)	9210 (194)	9333 (221)	10 574 (554)	13 701 (533)	13 061 (1018)	10 363 (194)
Informal care	11 220 (377)	5647 (606)	4433 (539)	9002 (293)	–	–	–	–	–
Total societal costs	18 800 (404)	16 221 (854)	17 816 (711)	18 211 (329)	–	–	–	–	–

**Appendix 3.** Six-month resource utilization estimates by care model from a societal perspective (a) and healthcare perspective (b).

(a) Societal perspective (including informal care)												
Service use category	CM1 n = 1331		CM2 n = 311		CM3 n = 418			Total N = 2060				
	Use of service, n (%)	Mean (SD)	Use of service, n (%)	Mean (SD)	Use of service, n (%)	Mean (SD)		Use of service, n (%)			Mean (SD)	
Home care												
Home health aide hours	675 (58%)	48.2 (87.4)	244 (91%)	114.4 (225.3)	294 (88%)	162.5 (172.5)		1213 (68%)			79.8 (142.9)	
Home nursing hours	560 (47%)	11.7 (23.6)	139 (53%)	24.3 (70.4)	268 (81%)	32.6 (34.8)		967 (55%)			17.5 (37.5)	
Physician visits												
Physician visits	780 (66%)	3.3 (4.5)	149 (56%)	2.9 (5)	325 (97%)	6.2 (4.5)		1254 (70%)			3.8 (4.7)	
Other healthcare services												
Physical therapy sessions	197 (17%)	5.7 (15.2)	63 (24%)	7.1 (15.1)	48 (14%)	5.2 (14.2)		308 (17%)			5.8 (15)	
Occupational therapy sessions	11 (1%)	0.2 (2.3)	0 (0%)	0 (0)	90 (30%)	15.1 (31.7)		110 (6%)			3 (15)	
Social worker sessions	8 (1%)	0.2 (3.7)	2 (1%)	0.1 (1.6)	2 (1%)	0.1 (1.5)		12 (1%)			0.2 (3.1)	
Hospital admissions												
Hospital admission with overnight stay, times	527 (45%)	0.8 (1.2)	82 (31%)	0.6 (1.2)	71 (21%)	0.3 (0.8)		680 (38%)			0.7 (1.1)	
Hospital admission with overnight stay, nights	527 (45%)	5.1 (7.8)	82 (31%)	3.7 (7.9)	71 (21%)	2.4 (5.9)		680 (38%)			4.4 (7.6)	
Emergency room visits without overnight stay	411 (35%)	0.6 (1.3)	49 (18%)	0.2 (0.6)	32 (10%)	0.1 (0.6)		492 (28%)			0.5 (1.1)	
Supportive care services												
Home making services hours	447 (38%)	23.1 (42.9)	128 (48%)	37.3 (50.7)	195 (58%)	26.7 (34.7)		770 (43%)			25.9 (43.1)	
Meals on wheels	311 (26%)	34 (63.2)	111 (41%)	57.3 (75.8)	119 (36%)	49.4 (72.1)		541 (30%)			40.4 (67.6)	
Institutionalized care												
Nursing home admissions	53 (4%)		11 (4%)		9 (2%)			73 (4%)				
Rehabilitation institute admissions	1 (0.1%)		0 (0%)		1 (0.2%)			2 (0.1%)				
Informal care												
Informal caregiver time (hours)	1073 (91%)	856 (998)	198 (74%)	372.1 (724.6)	172 (51%)	326.8 (763.7)		1443 (81%)			684 (951.5)	
(b) Healthcare perspective (without informal care)												
Service use category	CM1 n = 1716		CM2 n = 311		CM3 n = 439		CM4 n = 87			Total N = 2553		
	Use of service, n (%)	Mean (SD)	Use of service, n (%)	Mean (SD)	Use of service, n (%)	Mean (SD)	Use of service, n (%)		Mean (SD)	Use of service, n (%)		Mean (SD)
Home care												
Home health aide hours	822 (55%)	61.8 (110.1)	244 (91%)	114.4 (225.3)	307 (86%)	159.6 (171.1)	27 (39%)		62.8 (102.4)	1400 (64%)		84.3 (144.8)
Home nursing hours	858 (58%)	25.5 (40.4)	139 (53%)	24.3 (70.4)	288 (82%)	37.4 (43.6)	60 (100%)		68.1 (44.7)	1345 (63%)		28.5 (46.4)
Physician visits												
Physician visits	975 (65%)	3.9 (6.5)	149 (56%)	2.9 (5)	332 (94%)	6 (4.6)	42 (59%)		5.1 (6)	1498 (69%)		4.1 (6.1)
Other healthcare services												
Physical therapy sessions	273 (18%)	8.2 (22)	63 (24%)	7.1 (15.1)	59 (17%)	7.1 (18.8)	16 (22%)		18.5 (39.2)	411 (19%)		8.3 (21.7)
Occupational therapy sessions	34 (2%)	0.6 (4.9)	(0%)	0 (0)	109 (31%)	15.1 (31.1)	2 (3%)		1.4 (9.5)	145 (7%)		2.9 (14.3)
Social worker sessions	9 (1%)	0.2 (3.3)	2 (1%)	0.1 (1.6)	2 (1%)	0.1 (1.4)	(0%)		0 (0)	13 (1%)		0.2 (2.9)
Hospital admissions												
Hospital admission with overnight stay, times	581 (39%)	1.4 (7.1)	82 (31%)	0.6 (1.2)	76 (21%)	0.5 (2.8)	13 (18%)		3.4 (9.3)	752 (34%)		1.2 (6.3)
Hospital admission with overnight stay, nights	581 (39%)	4.8 (9.8)	82 (31%)	3.7 (7.9)	76 (21%)	2.5 (6.3)	13 (18%)		3.4 (9.3)	752 (34%)		4.3 (9.1)
Emergency room visits without overnight stay	423 (28%)	0.5 (1.2)	49 (18%)	0.2 (0.6)	33 (9%)	0.1 (0.6)	5 (7%)		0.1 (0.4)	510 (23%)		0.4 (1)
Supportive care services												
Home making services hours	598 (40%)	28.1 (46.8)	128 (48%)	37.3 (50.7)	207 (58%)	28.8 (37.5)	37 (53%)		60.9 (69.1)	970 (45%)		30.4 (47.2)
Meals on wheels	398 (26%)	34.5 (63.2)	111 (41%)	57.3 (75.8)	129 (36%)	49.5 (71.4)	17 (23%)		32.4 (62.5)	655 (30%)		39.6 (66.7)
Institutionalized care												
Nursing home admissions	71 (4%)		11 (4%)		10 (2%)		6 (7%)			98 (4%)		
Rehabilitation institute admissions	1 (0.1%)				1 (0.2%)					2 (0.1%)		
